# The need for Cre-loci controls in conditional mouse experiments: *Mrp8-cre transgene predisposes mice to antibody-induced arthritis*

**DOI:** 10.1038/s41435-024-00313-3

**Published:** 2024-12-04

**Authors:** Zhongwei Xu, Laura Romero‐Castillo, Àlex Moreno-Giró, Rajan Kumar Pandey, Rikard Holmdahl

**Affiliations:** Medical Inflammation Research, Division of Immunology, Department of Medical Biochemistry and Biophysics, Karolinska Institutet, Stockholm, Sweden

**Keywords:** Autoimmunity, Innate immunity

## Abstract

The Cre/loxP system is extensively utilized to pinpoint gene functions in specific cell types or developmental stages, typically without major disturbance to the host’s genome. However, we found that the random insertion of the *Mrp8-cre* transgene significantly promotes the host’s innate immune response. This effect is characterized by elevated susceptibility to cartilage antibody-induced arthritis, likely due to interference with genes near the insertion site. These findings underscore the potential biological disturbances caused by random transgene integration, and the necessity for stringent control strategies to avoid biased interpretations when using Cre-conditional strains.

## Introduction

Cartilage antibody-induced arthritis (CAIA) is a mouse model that mimics the effector phase of rheumatoid arthritis (RA). It utilizes arthritogenic antibody cocktails targeting joint proteins, typically inducing arthritis within days or even hours in susceptible strains [1]. This model bypasses adaptive immunity and directly activates the innate immunity, initiating inflammation by maximizing acute neutrophil expansion. Therefore, investigating neutrophil-specific gene expression patterns is essential to understand the onset of both mouse CAIA and human RA.

The Cre/loxP system has revolutionized medical research by enabling site-specific recombination, thus laying the groundwork for its widespread application [2]. This system allows for precise dissection of gene function within specific cells or developmental stages, effectively overcoming the limitations of conventional knockout strategies that disrupt genes universally. For instance, the *Mrp8-cre/ires-eGFP* transgenic mice (stock no: 021614), when crossed with mouse strains carrying loxP-flanked genes, can be utilized to manipulate these genes specifically expressed in granulocytes [3]. However, homozygous *Mrp8-cre*^*Tg/Tg*^ mice are nonviable [4], indicating the inherent toxicity of the inserted allele and its significant impact on the host’s biological systems. Therefore, using these mice necessitates careful characterization of the altered phenotypes and the implementation of stringent controls.

Here, we report that a single allele of the *Mrp8-cre/ires-eGFP* transgene predisposes mice to CAIA, likely due to the interfered expressions of genes close to the insertion site. These findings underscore the need for rigorous control strategies when using conditional mouse strains.

## Materials and methods

See Supplementary materials for details.

This study was performed according to the ethical permits (N35/16, 2660–2021) approved by the local animal ethics committee (Stockholm region) affiliated with the Swedish Board of Agriculture.

## Results

**Fig. 1:**
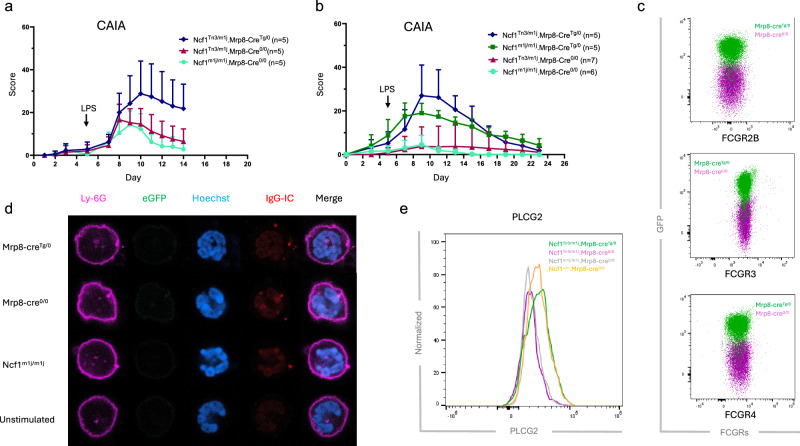
Mrp8-cre transgene predisposes mice to cartilage antibody-induced arthritis (CAIA). **a**, **b** Mice were immunized with cartilage antibody cocktail Cab4 containing M2139, ACC1, 15A, L10D9 on day 0 (3 mg, intravenously (i.v.)), and boosted with lipopolysaccharides (LPS) on day 5 (25 μg, intraperitoneally (i.p.)). **a** Ncf1^Tn3/m1j^.Mrp8-cre^Tg/0^ mice exhibited more severe arthritis compared to the other two control groups (Two-way ANOVA followed by multiple comparisons, *p* < 0.005 from d10 to d14); **b** Mrp8-cre^Tg/0^ mice with or without ROS restored exhibited more severe arthritis compared to the other two control groups (Two-way ANOVA followed by multiple comparisons, *p* < 0.001 between NCF1 mutated mice with or without Mrp8-cre transgenic allele from d7 to d15); **c** Neutrophils expressed mildly lower of FCGR2B, and comparable levels of FCGR3 and and FCGR4 in Mrp8-cre^Tg/0^ mice compared to WT littermates; **d** Neutrophils internalized comparable levels of immune complex (IC) in Mrp8-cre^Tg/0^, WT, and Ncf1^m1j/m1j^ mice; **e** Recombinant antibody R69-4 phosphorylated more phospholipase Cγ2 (PLCG2) in ROS sufficient neutrophils.

Our initial investigation aimed to elucidate the role of neutrophil-derived reactive oxygen species (ROS) in CAIA. To achieve this, we established mouse strains by crossing *Ncf1*^*Tn3/m1j*^ mice with *Ncf1*^*m1j/m1j*^*.Mrp8-cre*^*Tg/0*^ mice. Offspring harboring both *Ncf1*^*Tn3*^ and *Mrp8-cre*^*Tg*^ alleles exhibits restored ROS production exclusively in MRP8-expressing cells, mainly neutrophils, while littermates lacking either gene displayed uniformly low ROS levels across all cell subsets [5]. Following arthritogenic antibody cocktail transfer, *Ncf1*^*Tn3/m1j*^*.Mrp8-cre*^*Tg/0*^ mice (with ROS restored in neutrophils) displayed significantly more severe arthritis compared to *Ncf1*^*Tn3/m1j*^*.Mrp8-cre*^*0/0*^ or *Ncf1*^*m1j/m1j*^*.Mrp8-cre*^*0/0*^ littermates (Fig. 1a). These results seem in favor that neutrophil-derived ROS promotes arthritis development. However, there are two different variables within the experimental group that differ from the two control groups. First, *Ncf1*^*Tn3/m1j*^*.Mrp8-cre*^*Tg/0*^ mice have ROS production restored in neutrophils; Second, they harbor the *Mrp8-cre/ires-eGFP* transgene. Further investigation involving *Ncf1*^*m1j/m1j*^*.Mrp8-cre*^*Tg/0*^ mice revealed comparable arthritis severity to *Ncf1*^*Tn3/m1j*^*.Mrp8-cre*^*Tg/0*^ littermates (Fig. 1b). Conversely, littermates lacking the transgenic *Mrp8-Cre* allele exhibited milder arthritis (Fig. 1b). These results suggest that the presence of the *Mrp8-cre* transgene itself, rather than the restoration of ROS in neutrophils, predisposes mice to CAIA.

To determine whether this predisposition involves adaptive immune response apart from CAIA (which predominantly depends on innate immunity [6]), we employed a delayed-type hypersensitivity (DTH) model. DTH is a model for evaluating adaptive immune responses, primarily Th1 response [7]. After challenge, *Ncf1*^*m1j/m1j*^*.Mrp8-cre*^*0/0*^ mice exhibited comparable increase of ear thickness compared to *Ncf1*^*m1j/m1j*^*.Mrp8-cre*^*Tg/0*^ littermates (Fig. S1a). These results suggest that *Mrp8-cre*^*Tg/0*^ transgenic allele may not disturb adaptive immunity. Instead, its effect could be restricted in antibody-induced inflammation.

To investigate how the *Mrp8-cre* transgene disposes mice to CAIA, we measured the levels of FCGRs which are responsible for IgG-induced inflammation. The insertion of the *Mrp8-cre* transgene did not alter the expressions of any activating FCGRs on neutrophils but mildly lowered the expression of inhibitory FCGR2B (Fig. 1c, Fig. S1b). However, the internalization of IgG immune complexes (IC) by neutrophils was not affected (Fig. 1d). We then used a recombinant antibody, R69-4, known to phosphorylate neutrophil phospholipase Cγ2 (PLCG2) [8]. This phosphorylation mimics neutrophil activation upon IC stimulation through FCGRs. After a 10-min incubation, R69-4 phosphorylated more PLCG2 in ROS-sufficient neutrophils (both *Ncf1*^*Tn3/m1j*^*.Mrp8-cre*^*Tg/0*^ and *Ncf1*^*+/+*^*.Mrp8-cre*^*0/0*^ mice) (Fig. 1e), indicating that ROS, rather than the inserted *Mrp8-cre* transgene, plays a role in IC-mediated neutrophil activation.

**Fig. 2:**
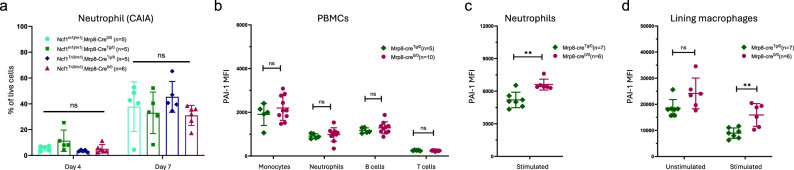
Mrp8-cre transgene compromises PAI-1 expression upon stimulation. **a** No significance was recorded regarding neutrophil proportion during arthritis development among the 4 genotypes (One-way ANOVA, *p* > 0.05); **b** No significance was recorded regarding PAI-1 expression on peripheral blood cells under steady state (Mann–Whitney *U* test, *p* > 0.05); **c** Mrp8-cre^Tg/0^ neutrophils exhibited compromised expression of PAI-1 upon stimulation compared to WT neutrophils (Mann–Whitney U test, *p* < 0.01); **d** No significance was recorded regarding PAI-1 expression on synovial lining macrophage between Mrp8-cre^Tg/0^ and WT neutrophils under steady state (Mann–Whitney U test, *p* > 0.05); Mrp8-cre^Tg/0^ synovial lining macrophages expressed significantly lower PAI-1 upon LPS stimulation compared to WT counterparts (Mann–Whitney *U* test, *p* < 0.01).

The above results suggest that *Mrp8-cre* insertion may not sensitize neutrophils upon IC stimulation at the priming stage. We then tracked neutrophil population during arthritis onset, and observed that neutrophil proportions (Fig. 2a) or absolute counts (Fig. S1c) in peripheral blood did not differ between *Mrp8-cre*^*Tg/0*^ and *Mrp8-cre*^*0/0*^ mice during either antibody phase or lipopolysaccharide (LPS) phase. Here it seems that *Mrp8-cre* transgene does not affect a typical pathophysiological procedure of antibody-induced arthritis.

Interestingly, Wang et al. [4] have demonstrated that the insertion of *Mrp8-cre* transgene disrupts two genes, *Ap1s1* and *Serpine1*. The absence of *Ap1s1* expression may underlie the preweaning lethality observed in homozygous *Mrp8-cre*^*Tg/Tg*^ mice, as *Ap1s1*^*-/-*^ mice fail to survive into adulthood [9] while *Serpine1*^*-/-*^ mice do survive [10]. Instead, the lack of *Serpine1* expression appears to be a more likely cause of the heightened susceptibility to CAIA compared to the lack of *Ap1s1*, because plasminogen activator inhibitor-1 (PAI-1), encoded by *Serpine1*, can indirectly inhibit the activity of matrix metalloproteinases [11] which dominates cartilage degradation. Furthermore, it has been shown that administration of PAI-1 suppresses septic arthritis by lowering plasmin-induced inflammatory responses and subsequent cartilage destruction [12].

Under steady state, we recorded significantly lower intracellular AP1S1 expression in *Mrp8-cre*^*Tg/0*^ neutrophils (Fig. S1d), but upon stimulation by LPS, *Mrp8-cre*^*Tg/0*^ neutrophils expressed equivalent levels of AP1S1 compared to *Mrp8-cre*^*Tg/0*^ neutrophils (Fig. S1d). In contrast, PAI-1 expression did not significantly differ between the two genotypes under steady state on any cell types from peripheral blood (Fig. 2b). However, once stimulated by LPS, *Mrp8-cre*^*Tg/0*^ neutrophils expressed significantly lower PAI-1 compared to WT littermates (Fig. 2c, Fig. S1e). Due to the universal disruption of *Serpine1* caused by *Mrp8-cre* insertion, irrespective of the transcription of *Mrp8* promoter in only granulocyte lineage, we proceeded to check PAI-1 expression in other cell types related to arthritis development, such as synovial lining macrophages. These macrophages (CD11b^+^CD68^+^CX3CR1^+^) expressed comparable PAI-1 in *Mrp8-cre*^*Tg/0*^ mice under steady-state conditions, but the difference became significant upon stimulation by LPS (Fig. 2d). Taken together, these results suggest that PAI-1 and AP1S1 expressions are affected by the insertion of *Mrp8-cre* transgene in different scenarios even in a heterozygous state, likely affecting all cell types.

## Discussion

In this study, we demonstrate that the *Mrp8-cre* transgene promotes CAIA in the corresponding transgenic mice. This effect might be due to the disruption of *Serpine1* and *Ap1s1* sequences where *Mrp8-cre* is inserted [4], leading to insufficient expressions of the two proteins in different scenarios. The mechanism of PAI-1 and AP1S1 on antibody-induced inflammation warrants further investigation.

Although precise genome editing technologies have expanded the application for creating genetically modified mice, transgenic mouse strains with random insertions of genes are still widely used. However, the random insertion of Cre genes could have unexpected impact on the host. Therefore, the integration site, potential disruption of regulatory elements, copy number variation, and chromosomal destabilization need to be carefully controlled. *Mrp8-cre/ires-eGFP* transgenic mice are a valuable tool for investigating functions of specific genes in granulocytes. However, the random insertion of the *Mrp8-cre* transgene has been shown to cause a 44-kb deletion of the host genome [4], and largely affect innate immunity, as demonstrated in this study. The disruption of *Serpine1* within this 44-kb sequence, leading to insufficient PAI-1 production, might be responsible for the increased susceptibility. PAI-1 principally inhibits plasminogen, and we have previously shown that functional plasminogen is needed for the development of collagen-induced arthritis [13]. Moreover, PAI-1 possesses pleiotropic functions in atherosclerosis, inflammation, fibrosis, and even cancer [14]. It is especially critical in neutrophil biology, including efferocytosis, activation, and influx, etc. [15–17]. In *Mrp8-cre* mice, the production of PAI-1 is not only compromised in neutrophils, but also in other cells like macrophages, further complicating the biology of this mouse strain.

Experimentally excluding the possible effects of putative phenotypes is challenging. In earlier studies using K/BxN serum transfer arthritis model, the presence of the *Mrp8-cre* transgenic allele alone did not significantly promote arthritis development, although a trend of increased paw swelling was recorded with only three *Mrp8-cre* mice included [18]. With the presently used CAIA model, the impact of the *Mrp8-cre* transgenic allele becomes discernible and significant (Fig. 1b). Moreover, the random insertion disrupts both *Serpine1* and *Ap1s1* genes, which unexpectedly has an effect even in a heterozygous state, highlighting the role of these two genes in impacting the development of antibody-induced arthritis. However, without specific *Serpine1* and/or *Ap1s1* knockout models, the causal relationship between these genes and immune regulation cannot be definitively established from our platform. Either *Serpine1*^*+/-*^ or *Ap1s1*^*+/-*^ mice showing similar phenotype compared to *Mrp8-cre*^*Tg/0*^ mice could enhance the likelihood of this putative causal relationship. Additionally, most of the experimental mice in this study exhibit low ROS levels due to NCF1 deficiency, which could result in different phenotypes under ROS-sufficient conditions.

In other strains carrying Cre recombinase, concerns have been raised in various contexts. For example, the *LysM-Cre* mouse line, where the *Cre* allele is inserted into the translational start site of the endogenous Lysozyme M (LysM) gene, was initially considered a suitable tool for manipulating myeloid cells [19]. However, it was later reported that most of the cells in the brain undergoing LysM-Cre-mediated recombination were neurons rather than myeloid cells [20]. Although *the Cx3cr1-CreERT2* strain is recommended for such cases [21], it has been noted that only a subset of resident macrophages expresses CX3CR1, limiting its utility for targeting all monocyte/macrophage lineage cells [22]. In contrast to these heterogeneous cell populations, mouse neutrophils predominantly express Ly6G, making it an ideal target for neutrophil-specific gene manipulation. The *Ly6G-Cre* mouse line, where Cre is inserted into the first exon of *Ly6g*, appears not to interfere with other genes or neutrophil function [23], and thus may be preferred over *Mrp8-Cre* mice for neutrophil-targeted studies. Even so, it remains critical to mandate proper control strategy when Cre transgenes are included in experimental settings. Littermates carrying Cre allele(s) but without loxP sites should always be included to balance the potential variability.

In conclusion, our observations underscore the vital importance of implementing proper control strategies in conditional animal research, such as using littermate control [24]. When mouse strains are genetically modified by random integration of transgenes, the inserted loci should be more carefully controlled.

## Supplementary information

Supplementary materials

## Data Availability

All data supporting the findings in this study are available from the corresponding author upon reasonable request.
