# Supplementary material for: The need for Cre-loci controls in conditional mouse experiments: Mrp8-cre transgene predisposes mice to antibody-induced arthritis
Source: Genes Immun. 2024 Dec 4;26(2):169–72. doi: 10.1038/s41435-024-00313-3 (PMC12006020; doi:10.1038/s41435-024-00313-3)
Supplement: Supplementary file 1 — Supplementary materials [file 41435_2024_313_MOESM1_ESM.pdf]

Supplementary materials

The need for Cre-loci controls in conditional mouse experiments: *Mrp8-cre transgene predisposes mice to antibody-induced arthritis*

Zhongwei Xu[1], Laura Romero-Castillo[1], Àlex Moreno-Giró[1], Rajan Kumar Pandey[1], Rikard Holmdahl[1]

[1] Medical Inflammation Research, Division of Immunology, Department of Medical Biochemistry and Biophysics, Karolinska Institutet

Correspondence to Prof. Rikard Holmdahl, rikard.holmdahl@ki.se

Supplementary Figures

Figure S1

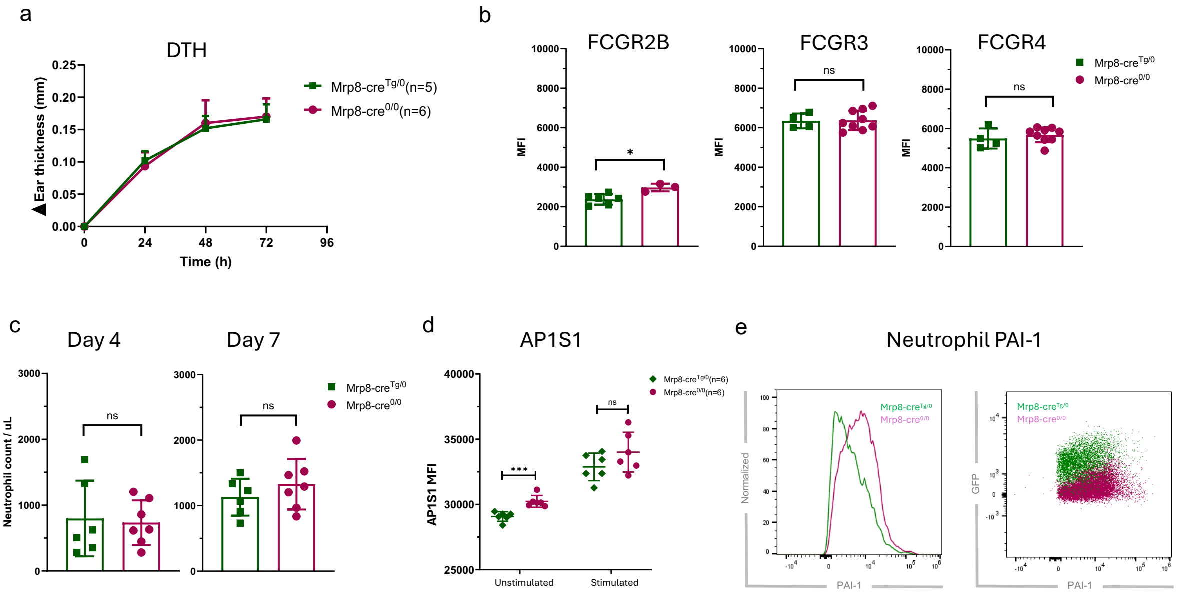

Figure S1. a. Increases of ear thickness did not differ in the delayed-type hypersensitivity (DTH) model between *Ncf1*$^{m1j/m1j}$*.Mrp8-cre*$^{Tg/0}$ mice and *Ncf1*$^{m1j/m1j}$*.Mrp8-cre*$^{0/0}$ littermates; **b**. *Ncf1*$^{m1j/m1j}$*.Mrp8-cre*$^{Tg/0}$ neutrophils express slightly lower FCGR2B and equivalent FCGR3 and FCGR4 compared to *Ncf1*$^{m1j/m1j}$*.Mrp8-cre*$^{0/0}$ littermates; **c**. Neutrophil absolute counts did not differ between the two genotypes on day 4 and day 7 of CAIA; **d**. Intracellular expression of AP1S1 was significantly lower in *Ncf1*$^{m1j/m1j}$*.Mrp8-cre*$^{Tg/0}$ mice at steady state, but became undiscernible upon stimulation; **e**. Representative flow cytometry plots showing the shift of PAI-1 expression in neutrophils between *Ncf1*$^{m1j/m1j}$*.Mrp8-cre*$^{Tg/0}$ mice and *Ncf1*$^{m1j/m1j}$*.Mrp8-cre*$^{0/0}$ littermates upon stimulation.

Supplementary Methods

Animals

The animals used in this study were housed in FELASA2 specific pathogen-free (SPF) facility in the Comparative Medicine's Annex (KM-A) at Karolinska Institute. *Ncf1*$^{m1j/m1j}$ mice carrying a naturally occurring lose-of-function mutation within the *Ncf1* region lack functional NCF1 expression, resulting in defective reactive oxygen species (ROS) production via the NADPH oxidase 2 (NOX2) complex [1]. *Ncf1*$^{Tn3/m1j}$ mice possess a floxed functional *Ncf1* allele, allowing the restoration of NCF1 expression upon recombination with Cre recombinase. Here we generated *Ncf1*$^{Tn3/m1j}$.*Mrp8-cre*$^{Tg/0}$ mice by crossing *Ncf1*$^{Tn3/m1j}$.*Mrp8-cre*$^{0/0}$ mice with *Ncf1*$^{m1j/m1j}$.*Mrp8-cre*$^{Tg/0}$ mice. This resulted in the restoration of ROS production in MRP8-expressing cells, primarily neutrophils [2]. Littermate control strategy [3] with intra-cage randomization was applied to minimize cage effect, and 3Rs principles [4] were applied when calculating the sample sizes.

Animal models

The cartilage antibody-induced arthritis (CAIA) model was established by injecting 3 mg of Cab4 cocktail (Vacara AB) containing M2139, ACC1, 15A, and L10D9 intravenously (*i.v.*) on day 0, and boosting with 25 μg of lipopolysaccharides (LPS) (Sigma Aldrich) intraperitoneally (*i.p.*) on day 5. Arthritis severity was scored according to a 60-point scale every 1-2 days by blinded investigators [5]. The delayed-type hypersensitivity (DTH) model was established by immunizing mice intradermally with 100 μg of bovine collagen type II (bCOL2) at the base of the tail. The injection volume was 100 μL, comprising a 1:1 emulsion of complete Freund's adjuvant (CFA, BD, Difco) and 10 mM acetic acid. On the eighth day following immunization, the right ear received an intradermal injection of 10 μL bCOL2 (1 mg/mL in acetic acid), while the left ear, serving as a control, was injected with 10 μL of acetic acid in PBS. Ear swelling was measured using a caliper at 0-, 24-, 48-, and 72-hours post-injection. The change in ear thickness was calculated by subtracting the swelling of the control ear from that of the bCOL2-injected ear and normalizing the results to the initial ear thickness measured on day 0 [6]. Mice (8 to 16 weeks old) included for CAIA were males and those for DTH were females.

Flow cytometry

Peripheral blood cells were obtained by bleeding mice from the left cheek, and 30 μL of blood was instantly diluted in 30 μL of PBS containing heparin. Red blood cells were lysed using ACK buffer twice (5 min each), and the remaining white blood cells were firstly stained using Fc block or fluorescent antibodies against FCGRs. These antibodies include unconjugated anti-CD16/32 (2.4G2), PE-Cy7 anti-CD16 (S17014E, Biolegend), APC anti-CD16.2 (9E9, Biolegend), and PerCP/Cy5.5 anti-CD32 (S17012B, Biolegend) antibodies in different experiments. Subsequently, cells were washed and stained using master mix containing antibodies against other targets. These antibodies include PE anti-CD45 (30-F11, Biolegend), Pacific blue anti-CD11b (M1/70, Biolegend), PerCP/Cy5.5 anti-CD3ε (500A2, Biolegend), APC anti-B220 (RA3-6B2, BD Biosciences), BV605 anti-Ly-6C (HK1.4, Biolegend), PE anti-Ly-6G (1A8, Biolegend), APC anti-F4/80 (BM8, Biolegend), FITC anti-CD68 (FA-11, Biolegend), PE anti-CX3CR1(SA011F11, Biolegend), FITC anti-CX3CR1(SA011F11, Biolegend), and PE/Cy7 anti-CX3CR1(SA011F11, Biolegend) antibodies in different experiments. Live/Dead Near IR dye (Thermo Fisher Scientific) was used to distinguished living cells from dead cells. Fluorescence with highly overlapping emission spectra to GFP (*Mrp8-cre* reporter fluorescence) was avoided to the most extent when neutrophils were expected in samples. All samples were protected from light throughout the procedures. After fluorescent staining, cells were analyzed based on the Attune NxT flow cytometer platform. Raw data from flow cytometry were analyzed using FlowJo program (BD Biosciences).

Regarding the measurement of PLCG2 phosphorylation, cells were incubated with R69-4 [7] (mIgG2b, 100 μg/mL) at 37 °C for 10 min. Following the incubation, cells were stained with master mix to discriminate cell populations. Subsequently, cells were fixed using 100 μL of Cytofix/CytoPerm solution (BD Biosciences) for 30 min, and permeabilized/stained using PE anti-PLCγ2 Phospho (Tyr759) (Biolegend) diluted in Perm/wash buffer (BD Biosciences) for 1 h. After the staining, cells were washed 3 times using the same Perm/wash buffer prior to flow cytometry analysis. In terms of AP1S1 staining, cells were similarly fixed, permeabilized, and stained with rabbit anti-mouse AP1S1 (polyclonal, Aviva Systems Biology) antibody diluted in Perm/wash buffer (BD Biosciences), followed

by labelling with PE donkey anti-rabbit IgG (polyclonal, Biolegend). Three times of washing were applied after staining. All staining procedures were protected from light.

Synovial macrophages were obtained by digesting hind paws (without skin or muscles) using RPMI media containing 2 mg/mL collagenase D (Sigma Aldrich) for 20 min at 37 °C. After digestion, cells were flushed out using RPMI media (Thermo Fisher Scientific) containing 10% FBS and subjected to fluorescent staining or *in vitro* stimulation. Regarding *in vitro* stimulation, neutrophils or macrophages were incubated with 500 ng/mL LPS for 1 h at 37 °C, and stained with mouse anti-PAI-1 antibody (1D5, Thermo Fisher Scientific) for 30 min, followed by incubation with goat-derived AF647 anti-mouse IgG-Fc antibody (polyclonal, SouthernBiotech) for another 30 min. Rat-derived fluorescent antibodies were subsequently used to discriminate cell populations.

Immunofluorescence

Immune complexes were made by incubating M2139 (500 µg/mL) and rat type-II collagen (250 µg/mL) at 4 °C overnight. Peripheral blood cells from different strains were then incubated with M2139 immune complexes (50 µg/mL) at 37 °C for 1 h. Following the incubation, cells were stained using PE anti-Ly6G (1A8, Biolegend) and Hoechst (Invitrogen) for 30 min. After the staining, cells were fixed, permeabilized, and intracellularly stained using AF647 anti-mouse IgG-Fc antibody (polyclonal, SouthernBiotech) for 1 h. Through rigorous wash, cells were finally visualized based on the Zeiss LSM800 confocal microscopy platform.

Statistical analyses

Statistical analyses were performed using GraphPad Prism (v10.1.2). For arthritis scores, two-way ANOVA followed by post hoc testing with Bonferroni or Dunnett correction was employed to determine the significances between groups. For quantitative data without assumed normal distribution, the Mann–Whitney U test was used to compare two groups. For independent measurements with more than 2 groups, one-way analysis of variance (ANOVA) followed by multiple comparisons with Bonferroni correction was employed for detecting significances. A two-sided $p < 0.05$ was considered as statistically significant.

References

1. Sareila O, Hagert C, Rantakari P, Poutanen M, Holmdahl R. Direct Comparison of a Natural Loss-Of-Function Single Nucleotide Polymorphism with a Targeted Deletion in the Ncf1 Gene Reveals Different Phenotypes. *PLOS ONE* 2015; **10**(11)**:** e0141974.

2. Zhong J, Li Q, Luo H, Holmdahl R. Neutrophil-derived reactive oxygen species promote tumor colonization. *Communications Biology* 2021; **4**(1)**:** 865.

3. Holmdahl R, Malissen B. The need for littermate controls. *European Journal of Immunology* 2012; **42**(1)**:** 45-47.

4. Sneddon LU, Halsey LG, Bury NR. Considering aspects of the 3Rs principles within experimental animal biology. *Journal of Experimental Biology* 2017; **220**(17)**:** 3007-3016.

5. Nandakumar KS, Holmdahl R. Efficient promotion of collagen antibody induced arthritis (CAIA) using four monoclonal antibodies specific for the major epitopes recognized in both collagen induced arthritis and rheumatoid arthritis. *Journal of Immunological Methods* 2005; **304**(1)**:** 126-136.

6. James J, Chen Y, Hernandez CM, Forster F, Dagnell M, Cheng Q *et al.* Redox regulation of PTPN22 affects the severity of T-cell-dependent autoimmune inflammation. *eLife* 2022; **11:** e74549.

7. Xu Z, Xu B, Lundström SL, Moreno-Giró À, Zhao D, Martin M *et al.* A subset of type-II collagen-binding antibodies prevents experimental arthritis by inhibiting FCGR3 signaling in neutrophils. *Nature Communications* 2023; **14**(1)**:** 5949.
